# Effect of aspirin on blood pressure in hypertensive patients: a systematic review and meta-analysis

**DOI:** 10.1186/s12872-024-03737-x

**Published:** 2024-02-06

**Authors:** Zehan Li, Shengchao Xu, Lin Chen, Danxian Jiang

**Affiliations:** Department of Head and Neck Oncology, Affiliated Hospital of Guangdong Medical University, Zhanjiang, 524001 Guangdong Province China

**Keywords:** Hypertension, Aspirin, Meta-analysis, Randomized controlled trial

## Abstract

**Introduction:**

Aspirin is widely used for secondary prevention in patients with hypertension. However, previous studies mainly focused on the preventive effects of aspirin, and there has been a lack of reliable evidence on whether taking aspirin affects blood pressure This study aimed to investigate whether aspirin would affect the blood pressure in patients with hypertension.

**Methods:**

PubMed, Cochrane database, Embase, Scopus and Medline databases were searched until September 2023. For continuous variables (e.g., blood pressure reduction), the mean difference (MD) was selected as the effect magnitude indices. We used the Cochrane Collaboration’s Risk of Bias tool to assess the risk of bias.

**Result:**

A total of five studies were included, comprising 20,312 patients. We found that aspirin did not affect SBP (MD = -0.78, 95% CI: − 2.41, 0.84). A similar result was found for DBP (MD = -0.86, 95% CI: − 2.14, 0.42).

**Conclusion:**

This study showed no significant difference in blood pressure between the aspirin and control groups, suggesting that aspirin does not affect blood pressure.

**Supplementary Information:**

The online version contains supplementary material available at 10.1186/s12872-024-03737-x.

## Introduction

Among patients diagnosed with hypertension, only 13.8% were considered controlled [1], and more than 9 million people die each year from hypertension-related diseases [2]. The most severe risk of hypertension is its complications, elevated systolic and diastolic blood pressures are strongly associated with cardiovascular disease risk [3, 4]. A follow-up study of 23,272 patients in the National Health and Nutrition Examination Survey (NHANES) showed that more than 50% of patients who died of coronary heart disease and stroke combined with hypertension [5], Population-based Atherosclerosis Risk in Communities (ARIC) study shows 25% of cardiovascular events are associated with hypertension [6]. However, despite a large number of hypertensive patients and the horrible complications it causes, the treatment of hypertension remains unsatisfactory. Unlike other diseases, hypertension has no apparent symptoms; in other words, hypertension is a so-called silent disease, so fewer patients will request treatment at an early stage or fail to follow prescriptions carefully [7]. Corrao et al. reported that more than 40% of patients would not continue initiating drug therapy within 1 year [8], about 10% of patients forget to take their daily medications [9]. It is therefore not surprising that exploring additional and complementary therapies for hypertension [10].

Aspirin is widely used for the secondary prevention of cardiovascular disease with positive effects [11–13]. In some secondary prevention studies, aspirin has been found to reduce the incidence of myocardial infarction and ischemic stroke [14–16]. However, previous studies mainly focused on the preventive effects of aspirin, and few studies have focused on the effects of aspirin on blood pressure [17]. There has been a lack of reliable evidence on whether taking aspirin affects blood pressure. Does it lower blood pressure and work in conjunction with other anti-hypertensive medications, or does it have no effect on blood pressure, or is it even more likely to cause fluctuations in blood pressure when taken over a long period? Some studies have found no relationship between aspirin and blood pressure [18–22], while Hermida et al. reported that taking aspirin at bedtime lowered blood pressure [23–28]. In their study, untreated hypertensive patients taking aspirin at bedtime reduced SBP and DBP by 6 mmHg and 4 mmHg, respectively. In conclusion, it remains uncertain what effect aspirin has on blood pressure, and therefore, studies are necessary to clarify the relationship between aspirin and blood pressure.

## Methods

This study was designed and carried out with the Preferred Reporting Items for Systematic Reviews and Meta-Analyses (PRISMA) criteria [29]. The protocol was registered in PROSPERO. (PROSPERO 2022 CRD42022346453).

### Eligibility criteria

Inclusion and exclusion criteria were based on population, interventions, comparisons, outcomes (PICO) criteria.

Studies meeting the following criteria will be included: (1) Participants should be at least 18 years old and diagnosed with hypertension (defined as systolic blood pressure greater than 140 mmHg or diastolic blood pressure greater than 90 mmHg, or both), their blood pressure was measured by validation techniques, receiving other anti-hypertensive medications was not restricted during the study period. (2) Participants in the intervention group should take aspirin, and the control group should take placebo or no treatment at all. (3) The study should report BP reduction as an outcome.

Studies with the following characteristics will be excluded: (1) Reviews, case reports and, conference abstracts. (2) Studies with less than 50 participants. (3) Literature for which experimental data were not available. (4) Articles for which full text was not available. (5) Non-clinical studies, such as in vivo or in vitro experiments. (6) Non-RCTs, including cohort studies and case-control trials. (7) Studies written in languages other than English.

After reading the title and abstract of the article, articles that met the inclusion criteria and did not conflict with the exclusion criteria will be read in full. Articles that had been read in full and meet the criteria will undergo data extraction. All publications were screened independently by two authors (Li, Xu), any disagreements were resolved after discussions between the two authors or by consulting a third reviewer.

### Search strategy

We searched the following databases for relevant papers: PubMed, Cochrane Library, Scopus, Embase, and Medline. We also searched the clinical research registry websites ClinicalTrials (clinicaltrials.gov) and ICTRP (trialsearch.who.int) to ensure no pertinent studies were missed. Searches were conducted up to September 2023.

### Data extraction

We utilized a data extraction form based on the Data Extraction Form of the Cochrane Review Group (Cochrane Collaboration) and modified some of its items to suit our study. The data extraction form mainly included the following: age, gender, race, country, blood pressure, intervention method, intervention duration, and primary outcomes. Two authors (Li, Xu) collected the data independently, and any disagreements were resolved through discussions between the two authors or by consulting a third reviewer.

### Quality assessment

As only RCTs were included, we used the Cochrane Collaboration’s Risk of Bias tool to assess study quality [30]. We used the Grading of Recommendations, Assessment, Development, and Evaluation (GRADE) criteria to assess the quality of evidence.

### Statistical analysis

We used Stata17 and Review Manager 5.4 software for data synthesis, with mean difference (MD) selected as the Effect Magnitude Indices for continuous variables (e.g., blood pressure reduction). For studies with more than one intervention group, we combined all intervention groups into one group as recommended by the Cochrane Handbook for the Systematic Review of Interventions [31]. Higgin’s I^2^ statistics and Cochran’s Q test were used to detect statistical heterogeneity between various studies [32]. When I^2^ > 50%, a random-effects model was used to evaluate the pooled results; otherwise, a fixed-effects model was used to analyze the pooled results. We used meta-regression to identify any possible sources of heterogeneity. A prespecified subgroup analysis was conducted according to [33] duration of intervention (>3 months vs. <3 months), time of aspirin administration (morning vs. evening), co-administration of other medications (co-administration of other medications vs. aspirin only), and type of control (placebo vs. no treatment). A sensitivity analysis was performed to assess the combined data’s stability and to pinpoint the cause of any heterogeneity. Begg’s and Egger’s linear regression tests were used to investigate publication bias [34].

## Result

### Study selection

**Fig. 1:**
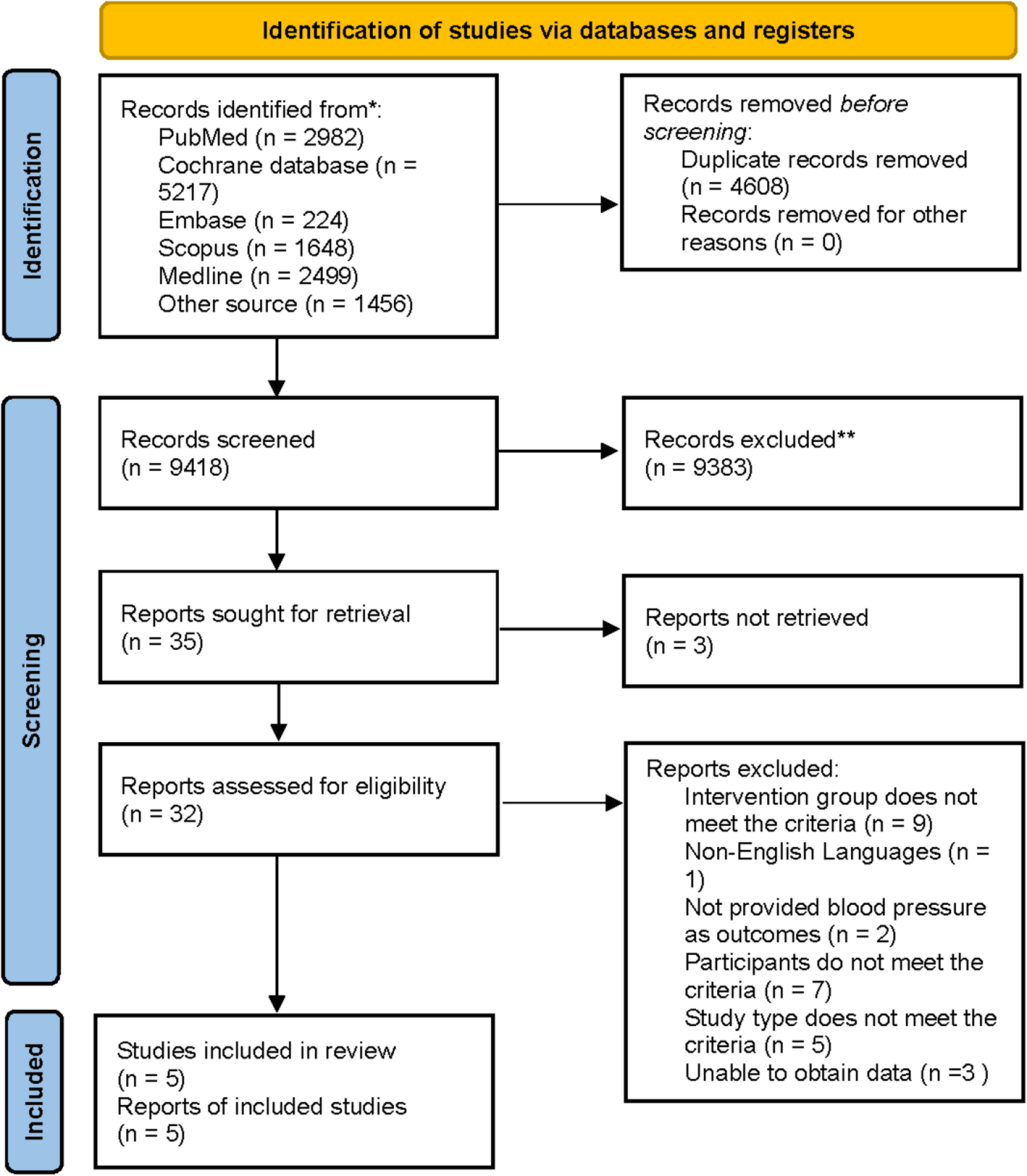
Flowchart of literature search and study selection

A total of 14,026 studies were identified through the database search. 4608 studies were removed due to duplication. After reading the titles and abstracts, 9383 articles were excluded. 30 articles were excluded after reading the full text for the following reasons: 3 studies could not obtain the full text, 9 were excluded because the intervention group did not meet the criteria, 1 was written in a language other than English, 2 did not provide blood pressure as an outcome, seven participants did not meet the criteria, 5 study type did not meet the criteria, 3 unable to obtain data. After that, 5 studies were included for analysis [22, 25, 35–37] (Fig. 1).

### Characteristics of the included studies

The characteristics of the eight included studies are shown in Table 1. A total of 20,312 participants were included. All participants were diagnosed with hypertension, mostly from high-income countries (HICs), such as Italy, Spain, and Poland. They were taking small doses of aspirin - usually less than or equal to 100 mg/day. Most studies had less than 3 months of follow-up. One study used placebo as a control; other studies did not have a treatment for the control group. One study prohibited participants from receiving other drugs concurrently, and four studies did not restrict.

**Table 1:** Characteristics of included studies

Study	No. of participants analyzed, (mean age ± SD, y), sex (male,%)	Types of control	Country	Dose	Follow up	Time to receive aspirin
Avanzini 2000	*N* = 142,(59 ± 5.9),53.5	no treatment	Italian	100 mg/day	3 months	awakening
Hermida 2003	*N* = 100,(42.5 ± 11.6),34	no treatment	Spain	100 mg/day	3 months	awakening/bedtime
hermida 2005	*N* = 328,(44.0 ± 12.6),34.5	no treatment	Spain	100 mg/day	3 months	awakening/bedtime
HOT 1998	*N* = 19,567, (61.5),53	placebo		75 mg/day	3·8 years (range 3·3–4·9 years)	awakening
Krasinska 2021	*N* = 175, (59.8),66.3	no treatment	Poland	75 mg/day	3 months	awakening/bedtime

### Risk of bias in included studies

**Fig. 2:**
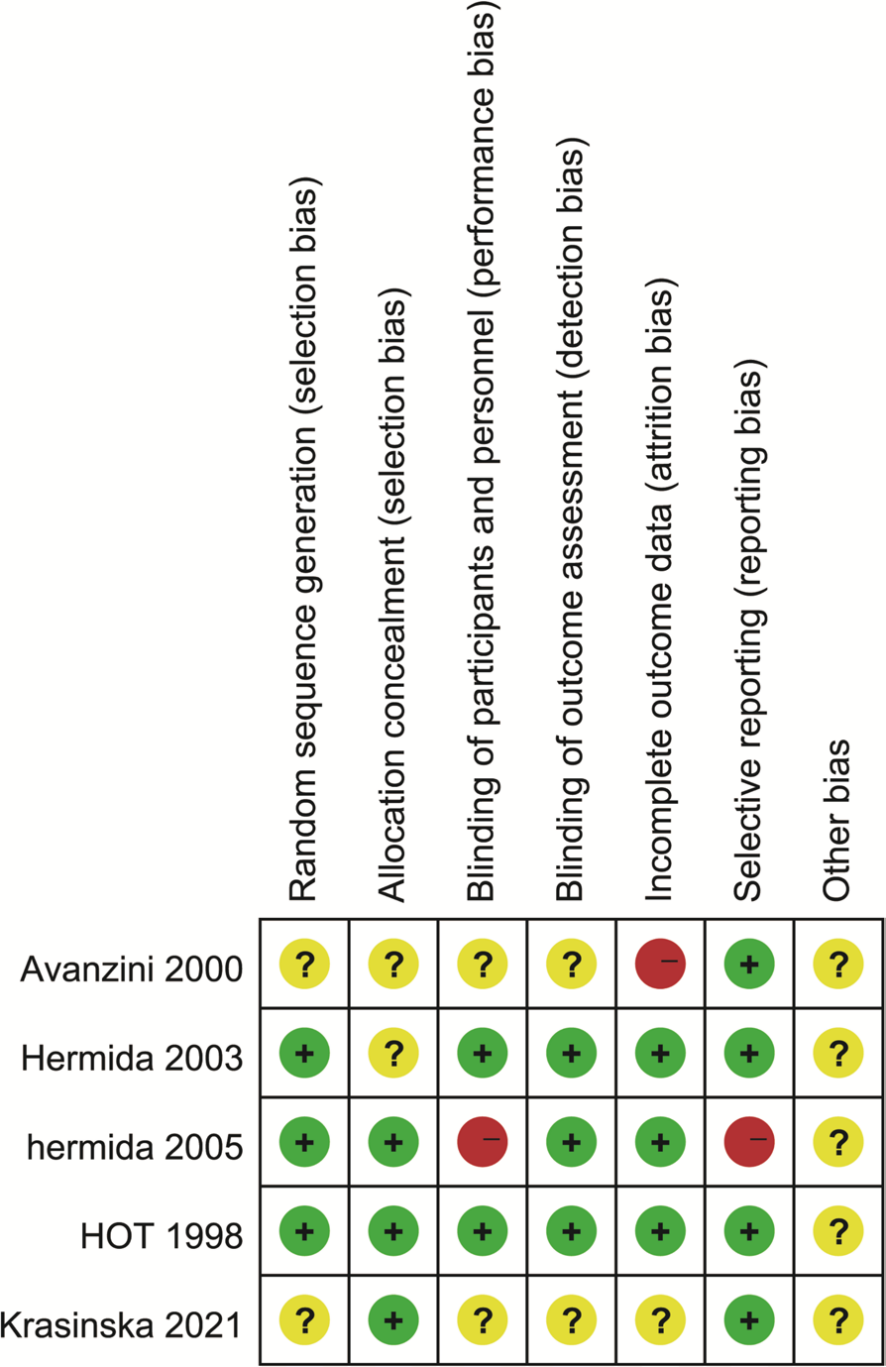
Risk of bias summary

We used the Cochrane Collaboration’s Risk of Bias tool to assess study quality, and the results are shown in Fig. 2. We considered the risk of bias unclear for studies where information supporting the judgment could not be found in the article. Avanzini et al. lost 14.5% of participants during the study, which may be a high risk for incomplete outcome data, especially considering that some of these individuals may quit the study due to side effects. One study was open-label, which we thought would lead to performance bias, so the risk of bias was set to high. One study did not report some of the outcome measures mentioned in the protocol, which we believed would create a high risk of reporting bias.

### BP reduction

**Fig. 3:**
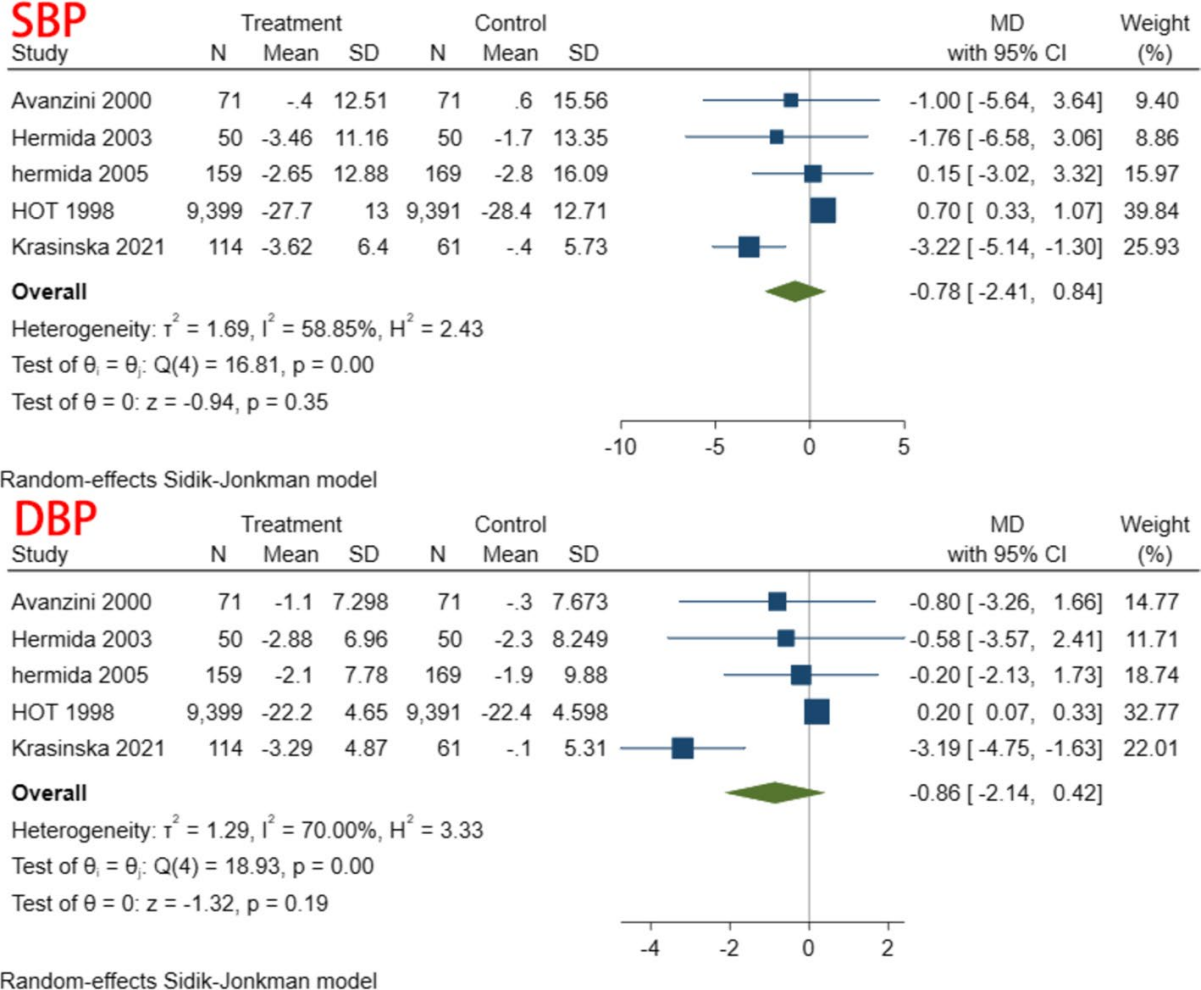
BP reduction

A total of 5 studies, including 20,312 individuals, were included in this study. Given the significant degree of heterogeneity (I^2^ = 58.85 and 70.00% for SBP and DBP, respectively), a random effects model was used. In terms of SBP, only one study found a significant decrease in SBP in patients treated with aspirin compared to controls [37]; the other studies showed no difference between the two groups, and the pooled results also suggest that aspirin does not affect SBP (MD = -0.78, 95% CI: − 2.41, 0.84). Similar result was found for DBP (MD = -0.86, 95% CI: − 2.14, 0.42). The forest plot of the synthesis results is shown in Fig. 3. The data are shown in Table 2. The GRADE ratings for both outcomes are low (Table 3).

**Table 2:** BP reduction between aspirin and control groups

BP	Studies	MD and 95% CI	Effects model	Heterogeneity (I^2^)
SBP	5	-0.78(−2.41, 0.84)	Random	58.85%
DBP	5	-0.86(−2.14, 0.42)	Random	70.00%

I^2^ with 95% CI for SBP: 0.458705836–0.907021197

I^2^ with 95% CI for DBP:0.465556395–0.907804755

**Table 3:** Grading of recommendations, assessment, development and evaluations (GRADE) criteria for outcomes

Quality assessment							No. of patients		Effect		Quality	Importance
No. of studies	Design	Risk of bias	inconsistency	Indirectness	Imprecision	Other Considerations	Aspirin	Control	Relative (95% CI)	Absolute		
SBP												
5	Randomised trials	No serious risk of bias	Serious^1^	No serious indirectness	Serious^2^	none	9793	9742	–	MD 0.10 lower (0.3 lower to 0.1 higher)	⊕ ⊕ ○○LOW	IMPORTANT
DBP												
5	Randomised trials	No serious risk of bias	Serious^3^	No serious indirectness	Serious^2^	none	9793	9742	–	MD 0.14 lower (0.37 lower to 0.09 higher)	⊕ ⊕ ○○LOW	IMPORTANT

^1^Heterogeneity = 58.85%

^2^Meets OIS criteria, but 95% CIs include null effect line

^3^Heterogeneity = 70.00%

### Meta-regression

Since it can be considered a source of heterogeneity when the *P*-value is less than 0.05, we may infer that different administration times may be a source of significant heterogeneity. (*P* = 0.051, 0.001, respectively) (Table 4A). According to the meta-regression results, the remaining various may not be a source of heterogeneity in this study (*p* = 0.320,0.376 for control, Table 4B; *p* = 0.628,0.638 for medication, Table 4C; *p* = 0.320,0.376 for duration, Table 4D).

**Table 4:** Meta regress for time(A) control(B) drug(C) and duration(D)

	_meta_es	Coefficient	Std. err.	z	*P* > |z|	[95% conf. interval]	
A.							
SBP	time	−.3544133	.1816568	− 1.95	0.051	−.7104541	.0016274
	_cons	.3421825	.256046	1.34	0.181	−.1596584	.8440234
DBP	time	−.3197575	.1005632	−3.18	0.001	−.5168577	−.1226572
	_cons	.3618581	.1036039	3.49	0.000	.1587983	.564918
B.							
SBP	control	.2241015	.2255029	0.99	0.320	−.2178762	.6660791
	_cons	−.3937554	.3099393	−1.27	0.204	−1.001225	.2137145
DBP	control	.2480603	.2799283	0.89	0.376	−.3005891	.7967098
	_cons	−.4528708	.3746845	−1.21	0.227	− 1.187239	.2814973
C.							
SBP	drug	.165251	.3410547	0.48	0.628	−.5032039	.8337059
	_cons	−.3202675	.4543974	−0.70	0.481	−1.21087	.5703351
DBP	drug	.189433	.4024724	0.47	0.638	−.5993985	.9782644
	_cons	−.4012255	.5356204	− 0.75	0.454	−1.451022	.6485711
D.							
SBP	duration	.2241015	.2255029	0.99	0.320	−.2178762	.6660791
	_cons	−.3937554	.3099393	−1.27	0.204	−1.001225	.2137145
DBP	duration	.2480603	.2799283	0.89	0.376	−.3005891	.7967098
	_cons	−.4528708	.3746845	−1.21	0.227	−1.187239	.2814973

### Subgroup analysis

**Fig. 4:**
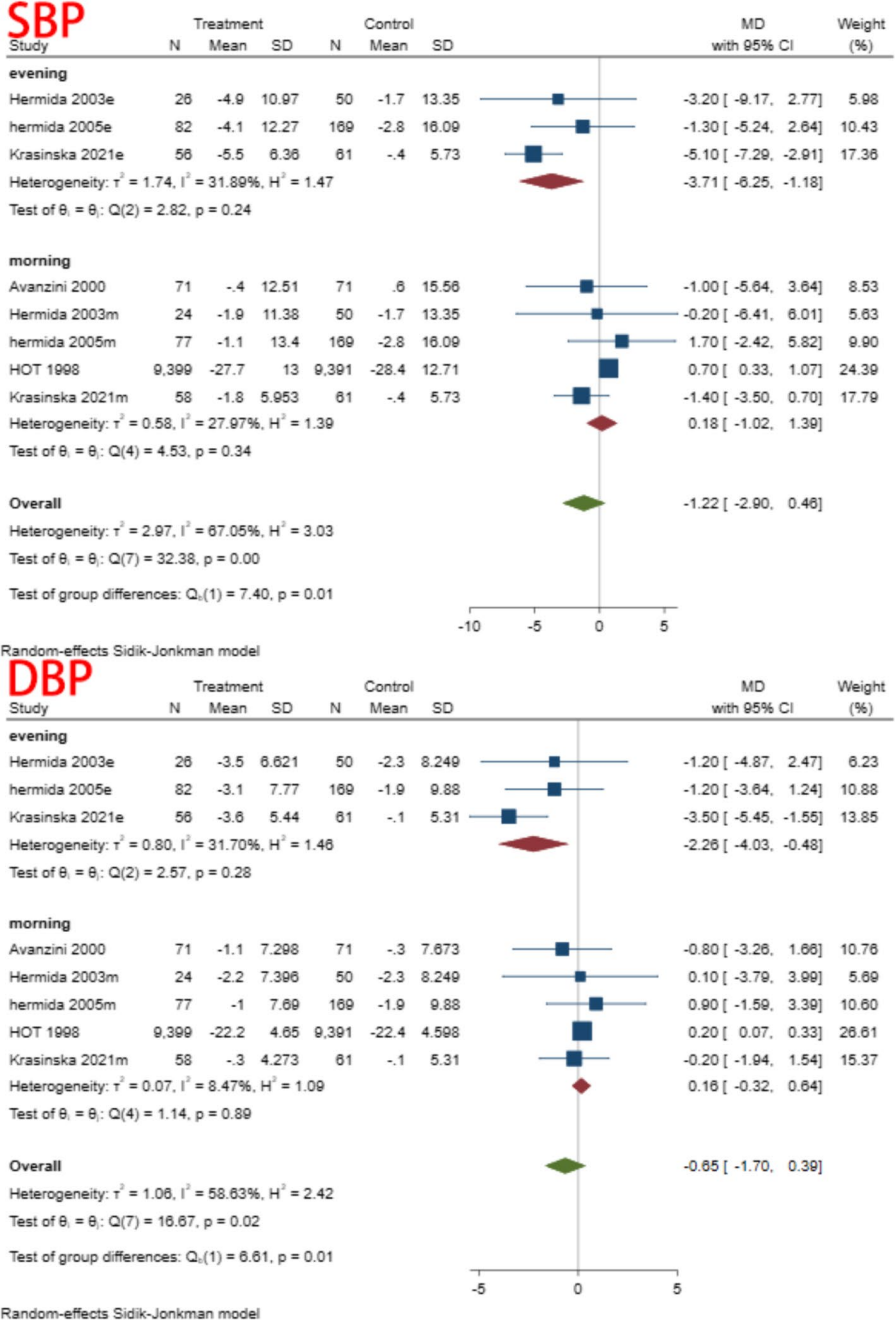
Subgroup analysis of administration time

**Fig. 5:**
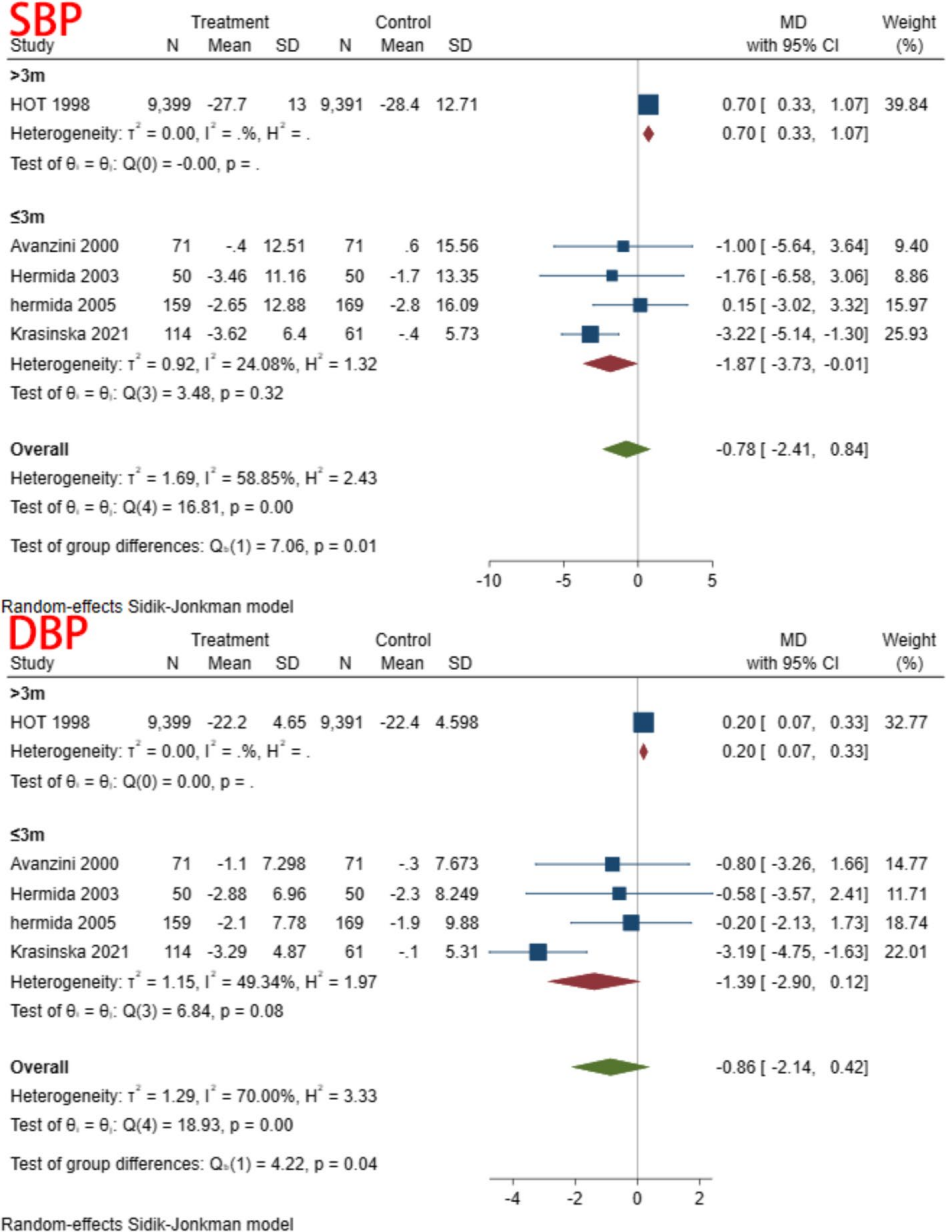
Subgroup analysis of follow up

**Fig. 6:**
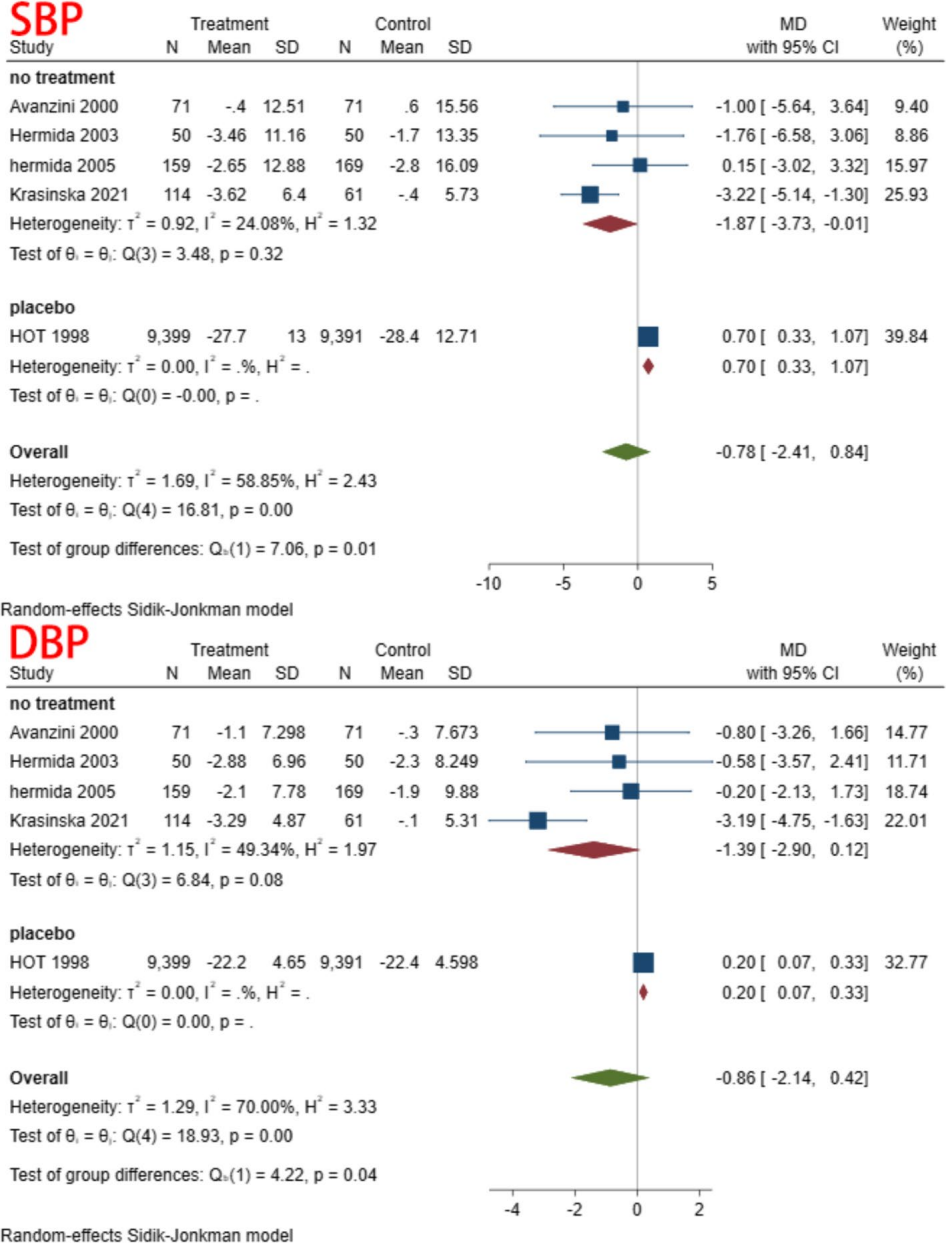
Subgroup analysis of types of control

**Fig. 7:**
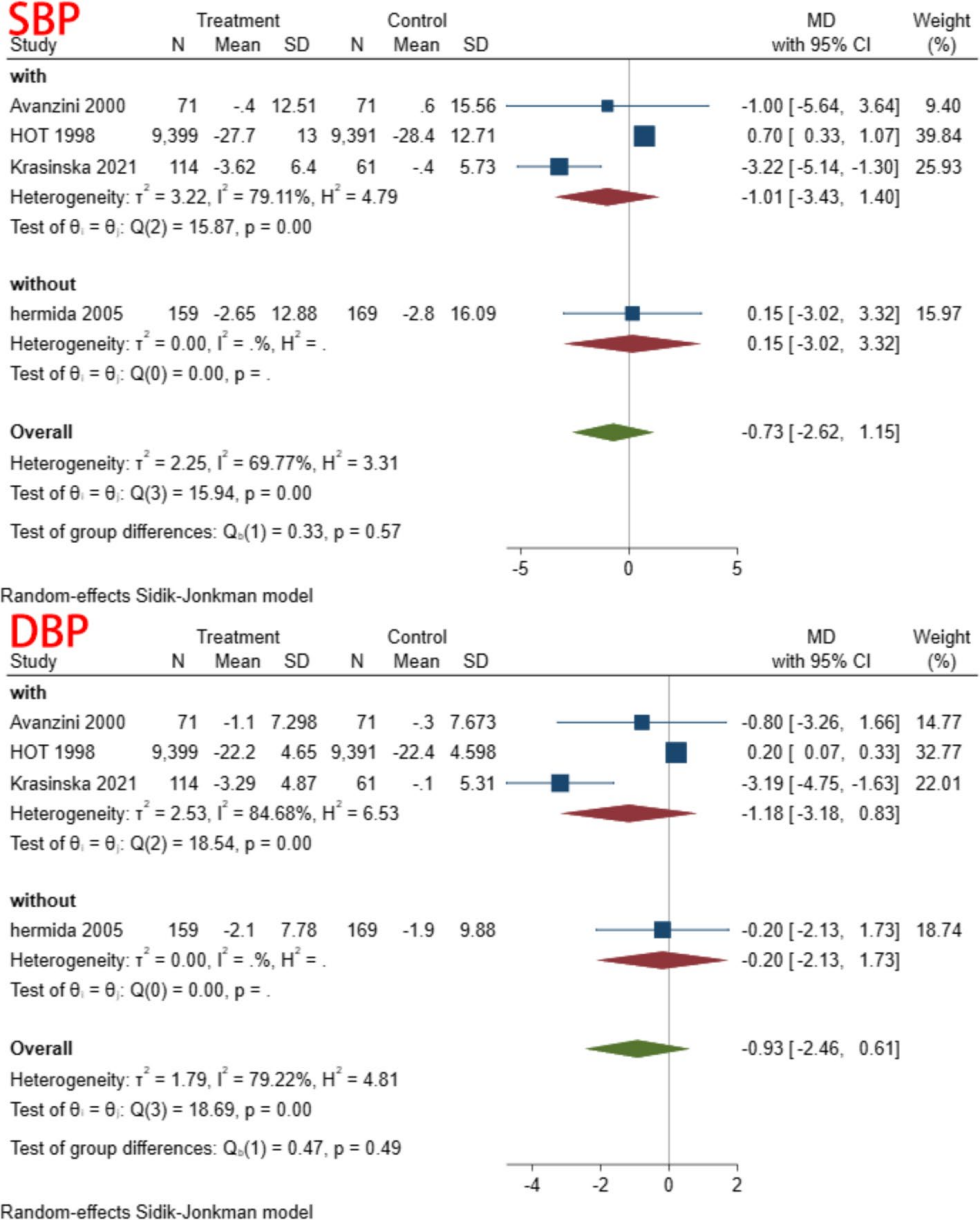
Subgroup analysis of receive other drug

A subgroup analysis was conducted based on time of aspirin administration (morning vs. evening), duration of intervention (>three months vs. <three months), co-administration of other medications (co-administration vs. aspirin only), and type of control (placebo vs. no treatment). In the subgroup analysis of different administration times, we found heterogeneity decreased in both subgroups. This result suggested that dosing time may be one of the reasons for the heterogeneity (Fig. 4) (Table 5). There were no significant differences in the remaining subgroups (Figs. 5, 6 and 7) (Table 6, 7 and 8).

**Table 5:** Subgroup analysis of time to receive aspirin

Subgroup	Studies	MD and 95% CI	Effects model	Heterogeneity (I^2^)
SBP				
Evening	3	−3.71(−6.25, − 1.18)	Random	31.89%
Morning	5	0.18(−1.02,1.39)	Random	27.97%
DBP				
Evening	3	−2.26(−4.03,-0.48)	Random	31.70%
Morning	5	0.16(−0.32,0.64)	Random	8.47%

**Table 6:** Subgroup analysis of follow up

Subgroup	Studies	MD and 95% CI	Effects model	Heterogeneity (I^2^)
SBP				
> 3 m	1	0.70(0.33,1.07)	Random	
< 3 m	4	−1.87(−3.73,−0.01)	Random	24.08%
DBP				
> 3 m	1	0.20(0.07,0.33)	Random	
< 3 m	4	−1.39(−2.90,0.12)	Random	49.34%

**Table 7:** Subgroup analysis of types of control

Subgroup	Studies	MD and 95% CI	Effects model	Heterogeneity (I^2^)
SBP				
No treatment	4	−1.87(−3.73,-0.01)	Random	24.08%
Placebo	1	0.70(0.33,1.07)	Random	
DBP				
No treatment	4	−1.39(−2.90,0.12)	Random	49.34%
Placebo	1	0.20 (0.07,0.33)	Random

**Table 8:** Subgroup analysis of receive other drug

Subgroup	Studies	MD and 95% CI	Effects model	Heterogeneity (I^2^)
SBP				
With	3	−1.01(−3.43,1.40)	Random	79.11%
Without	1	0.15(−3.02, 3.32)	Random	
DBP				
With	3	−1.18(−3.18,0.83)	Random	79.22%
Without	1	−0.20 (−2.13,1.73)	Random	

### Publish bias test

**Fig. 8:**
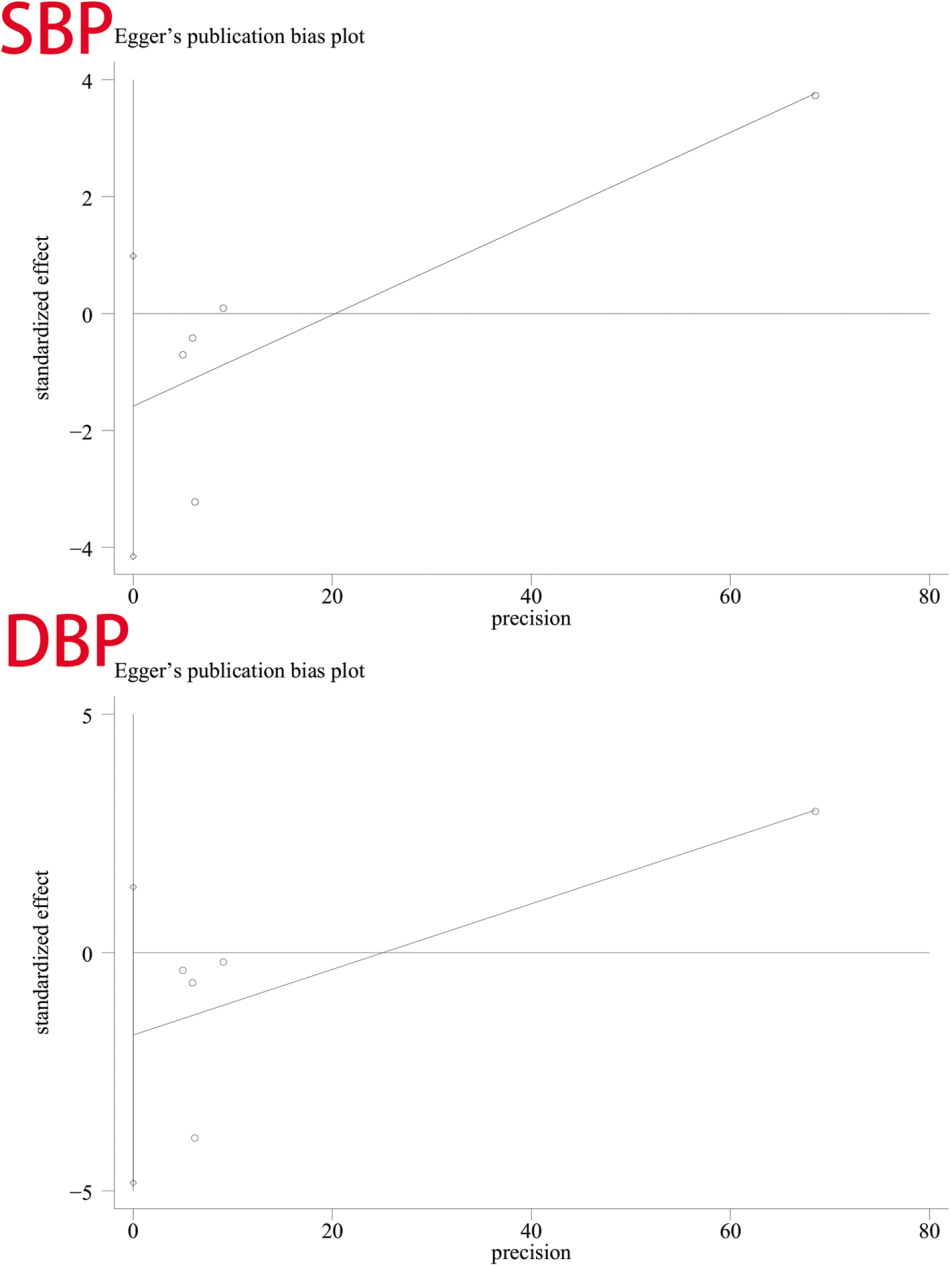
Egger’s test for SBP and DBP

**Fig. 9:**
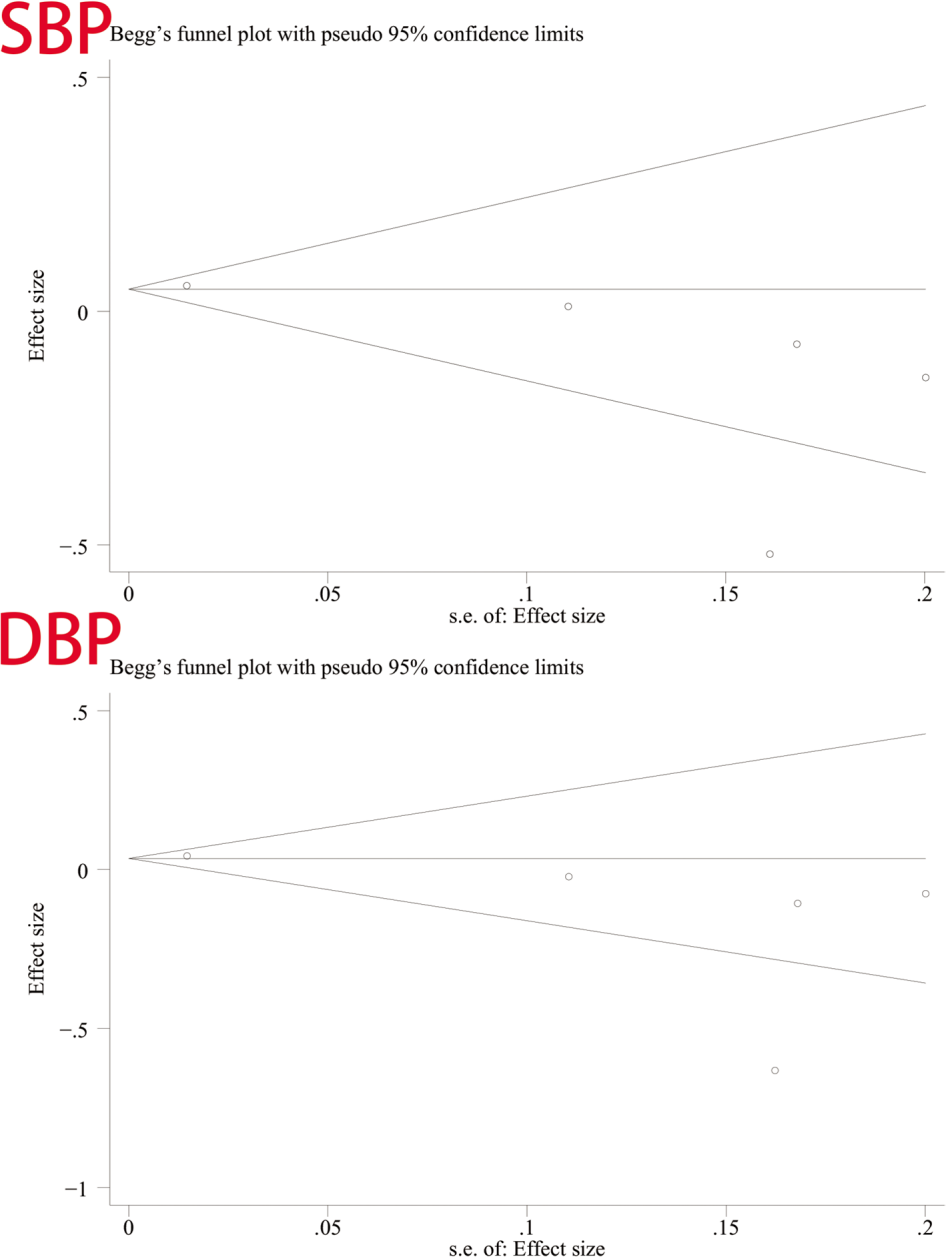
Begg’s funnel plot for SBP and DBP

We used Begg’s and Egger’s linear regression tests to detect publication bias (Table 9). The results of Egger’s test and Begg’s test are shown in Figs. 8 and 9. No asymmetry was observed in the funnel plot. According to Egger’s test, no publication bias was observed (*P* = 0.145, *P* = 0.174). A similar conclusion can be derived from Begg’s test (*P* = 0.221, *P* = 0.462).

**Table 9:** Begg test and Egger test

Blood Pressure	Begg’s test		Egger’s test		
	z	p	t	p	95% CL
SBP	1.22	0.221	−1.96	0.145	−4.152124, 0.9845658
DBP	0.73	0.462	−1.78	0.174	−4.833121, 1.369511

### Analysis of sensitivity

**Fig. 10:**
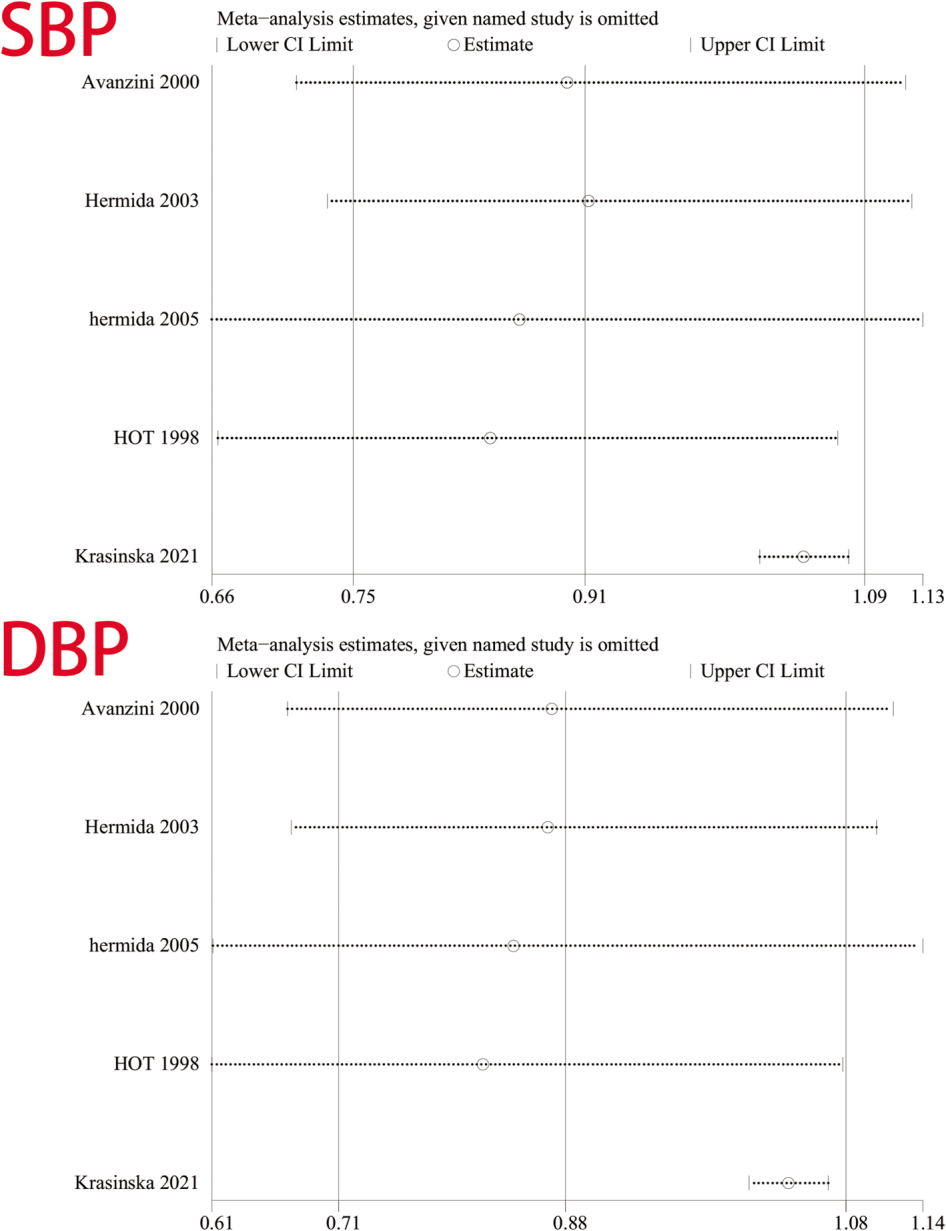
Sensitivity analysis for SBP and DBP

We found that removing studies one by one did not significantly change the results, suggesting that the overall results were not influenced by individual studies. The results for SBP ranged from 0.84 (95% CI: 0.66–1.08) to 1.05 (95% CI: 1.02–1.08), and for DBP from 0.81 (95% CI: 0.61–1.08) to 1.04 (95% CI: 1.01–1.07) (Fig. 10).

## Discussion

Some hypertensive patients need to take aspirin for a long time to prevent cardiovascular events, so it is natural to wonder whether aspirin affects blood pressure and the effect of antihypertensive drugs. Aspirin blocks TXA2 and promotes nitric oxide synthesis, which may lead to vasodilation, reduced peripheral vascular resistance, and lower blood pressure [36]. Some studies have also found that aspirin inhibits COX-1 and COX-2. Inhibition of COX-1 leads to vasoconstriction, which in turn leads to increased blood pressure [38]. In addition, some studies have shown that different dosing times or drug interactions can also affect blood pressure [28, 39, 40]. Because of conflicting findings from previous studies, we conducted this meta-analysis and systematic review to clarify what effect aspirin would have on patients with hypertension.

Our study found that aspirin did not change blood pressure levels, either SBP (MD = -0.78, 95% CI: − 2.41, 0.84) or DBP (MD = -0.86, 95% CI: − 2.14, 0.42). We found no significant difference between subgroups after subgroup analysis for factors such as administration time, follow-up, type of control, and concomitant treatment with other medications. However, caution should be exercised in interpreting the findings due to the few included studies. Although some studies have reported that different administration times, especially at bedtime, can lower blood pressure [27, 28], our study showed no significant difference between taking aspirin at bedtime and in the morning. However, the pooled results for the different subgroups do show contrasting trends, and perhaps with more studies included, this difference will be statistically significant. Patients with hypertension often have other co-morbidities that require them to take multiple medications simultaneously. Subgroup analysis showed that concomitant administration of other drugs does not change the result, which could indicate that aspirin does not interfere with other medications, which is consistent with the findings of Johnson et al. [41]. The rest of the subgroup analyses also showed no significant differences between groups. Begg’s test and Egger’s test showed no significant publication bias. However, the publication bias test is not very reliable due to the small number of included studies [34]. Sensitivity analysis showed that the pooled results were not affected by any single studies, and after removing individual studies, the conclusions remained consistent with the main study findings.

In fact, despite some controversy, most people agree that aspirin does not affect blood pressure [20, 21, 42, 43], and our study simply provides more compelling evidence to dispel some of the concerns in clinical practice. Aspirin, primarily used as an antiplatelet drug, may affect blood pressure through certain pathways. For example, it can inhibit the synthesis of the vasodilators PGI2 and PGE2 by blocking COX-1 and COX-2 and causing sodium and water retention [44]. Aspirin can also act in the vascular endothelium to inhibit the expression of pro-inflammatory cytokines and the adhesion of leukocytes, increasing the generation of nitric oxide, which ultimately leads to vasodilatation. This effect becomes more potent as thromboxane A2 and prostaglandins decrease [45–47]. We hypothesized that perhaps it is this delicate balance that led to the results of our study, namely that taking aspirin did not affect blood pressure. On the other hand, the effects mentioned above are limited and indirect because aspirin cannot directly affect the mechanisms that regulate blood pressure, which may explain why no blood pressure fluctuations were observed in patients taking aspirin. Our findings are also consistent with some recent studies; for example, in the study by Mirabito Colafella et al., they found that aspirin does not affect vascular function [48]. In a study by Dong et al., they found that aspirin has no significant effect on the gut microbiota of spontaneously hypertensive rats, and alterations in the gut microbiota may be associated with hypertension [49].

Subgroup analyses of different dosing times showed no significant difference in the antihypertensive effect when administered in the morning versus in the afternoon or at bedtime. However, due to the small number of participants in the bedtime dosing subgroup, this conclusion may not be as reliable, especially since some studies did propose that different dosing times result in different antihypertensive effects [50]. Since the secretion of nitric oxide, prostaglandins, angiotensin II, and angiotensin-converting enzyme has a circadian rhythm, different administration times may produce different effects [51, 52]. However, whether these differences are sufficient to alter blood pressure levels remains uncertain. Therefore, we believe there is a need for more trials focusing on this variable.

## Limitations

This study also has some limitations. First, most of the included studies had small sample sizes or low study quality, which would lead to unstable and less credible results. Second, the number of included studies was also small, so the assessment of publication bias may lack accuracy, and the overall results may also lack statistical significance. Finally, most of the included studies did not use placebo as a control. Since 20–24% of long-term changes in blood pressure are attributable to the placebo effect [53], the lack of placebo may cause a misinterpretation of the study results.

### Future directions

Our findings reduce some of the uncertainty in clinical practice and the process of developing guidelines. Clinicians may not have to worry about whether prescribing aspirin to patients with controlled hypertension will cause blood pressure fluctuations. As aspirin is widely used for secondary prevention of cardiovascular disease, this study may lead to updates in some guidelines. In conclusion, our systematic review and meta-analysis summarizes the results of global studies and draws compelling conclusions about whether aspirin affects blood pressure.

## Conclusion

This meta-analysis investigated whether taking aspirin affects blood pressure. The results showed no significant difference in blood pressure between the intervention and control groups, suggesting that aspirin does not lower or raise blood pressure. However, more studies are needed to confirm these findings.

### Supplementary Information

**Additional file 1.**

**Additional file 2.**

## Data Availability

The datasets used and/or analyzed during the current study are available from the corresponding author upon reasonable request.

## Abbreviations

NSAIDs: Nonsteroidal anti-inflammatory drugs
COX: Cyclooxygenase
PG: Prostaglandin
TXA2: Thromboxane A2
BP: Blood pressure
RCT: Randomized controlled trial
MD: Mean difference
CI: Confidence intervals
