# Supplementary material for: Effect of aspirin on blood pressure in hypertensive patients: a systematic review and meta-analysis
Source: BMC Cardiovasc Disord. 2024 Feb 6;24:90. doi: 10.1186/s12872-024-03737-x (PMC10845465; doi:10.1186/s12872-024-03737-x)
Supplement: Supplementary file 1 — Additional file 1. [file 12872_2024_3737_MOESM1_ESM.docx]

| Search | PubMed |
| --- | --- |
| #1 | "Hypertension"[Mesh] |
| #2 | antihypertens*[Title/Abstract] OR hypertens*[Title/Abstract] OR prehypertens*[Title/Abstract] |
| #3 | "Blood Pressure"[Mesh] |
| #4 | blood pressur*[Title/Abstract] OR bloodpressur*[Title/Abstract] |
| #5 | arterial pressur*[Title/Abstract] OR diastolic pressur*[Title/Abstract] OR systolic pressur*[Title/Abstract] |
| #6 | BP[Title/Abstract] OR DBP[Title/Abstract] OR SBP[Title/Abstract] |
| #7 | 1 OR 2 OR 3 OR 4 OR 5 OR 6 |
| #8 | "Aspirin"[Mesh] |
| #9 | acetylsalicylic acid[Title/Abstract] |
| #10 | aspirin[Title/Abstract] |
| #11 | ASA[Title/Abstract] |
| #12 | 8 OR 9 OR 10 OR 11 |
| #13 | preeclamp*[Title/Abstract] OR eclamp*[Title/Abstract] OR pregnan*[Title/Abstract] |
| #14 | ocular[Title/Abstract] OR portal[Title/Abstract] OR pulmonary[Title/Abstract] |
| #15 | 13 OR 14 |
| #16 | 7 AND 12 NOT 15 |
|  |  |
| Filter | ("Randomized Controlled Trial" [Publication Type] OR "Controlled Clinical Trial" [Publication Type] OR "Clinical Trials as Topic"[Mesh:NoExp] OR randomized[Title/Abstract] OR placebo [Title/Abstract] OR randomly[Title/Abstract] OR trial[Title/Abstract]) NOT ("Animals"[Mesh] NOT "Humans"[Mesh]) |
